# Age-related brain atrophy is not a homogenous process: Different functional brain networks associate differentially with aging and blood factors

**DOI:** 10.1073/pnas.2207181119

**Published:** 2022-12-02

**Authors:** Nikola T. Markov, Cutter A. Lindbergh, Adam M. Staffaroni, Kevin Perez, Michael Stevens, Khiem Nguyen, Natalia F. Murad, Corrina Fonseca, Judith Campisi, Joel Kramer, David Furman

**Affiliations:** ^a^Buck AI Platform, Buck Institute for Research on Aging, Novato, CA 94945; ^b^Department of Neurology, Memory and Aging Center, University of California San Francisco, Weill Institute for Neurosciences, San Francisco, CA 94158; ^c^Department of Psychiatry, University of Connecticut School of Medicine, Farmington, CT 06030; ^d^University of Lausanne, Lausanne CH-1015, Switzerland; ^e^Nguyen Tat Thanh Hi-Tech Institute, Nguyen Tat Thanh University, Ho Chi Minh City 70000, Vietnam; ^f^Instituto de Investigaciones en Medicina Traslacional, Universidad Austral, Consejo Nacional de Investigaciones Científicas y Técnicas, Pilar 1629, Argentina; ^g^Stanford 1000 Immunomes Project, Stanford University School of Medicine, Stanford, CA 94305

**Keywords:** brain aging, cytokines, cytokine clock, aging, gray matter volume

## Abstract

Aging is characterized by a progressive loss of brain volume at an estimated rate of 5% per decade after age 40. While these morphometric changes, especially those affecting gray matter and atrophy of the temporal lobe, are predictors of cognitive performance, the strong association with aging obscures the potential parallel, but more specific role, of individual subject physiology. Here, we studied a cohort of 554 human subjects who were monitored using structural MRI scans and blood immune protein concentrations. Using machine learning, we derived a cytokine clock (CyClo), which predicted age with good accuracy (Mean Absolute Error = 6 y) based on the expression of a subset of immune proteins. These proteins included, among others, Placenta Growth Factor (PLGF) and Vascular Endothelial Growth Factor (VEGF), both involved in angiogenesis, the chemoattractant vascular cell adhesion molecule 1 (VCAM-1), the canonical inflammatory proteins interleukin-6 (IL-6) and tumor necrosis factor alpha (TNFα), the chemoattractant IP-10 (CXCL10), and eotaxin-1 (CCL11), previously involved in brain disorders. Age, sex, and the CyClo were independently associated with different functionally defined cortical networks in the brain. While age was mostly correlated with changes in the somatomotor system, sex was associated with variability in the frontoparietal, ventral attention, and visual networks. Significant canonical correlation was observed for the CyClo and the default mode, limbic, and dorsal attention networks, indicating that immune circulating proteins preferentially affect brain processes such as focused attention, emotion, memory, response to social stress, internal evaluation, and access to consciousness. Thus, we identified immune biomarkers of brain aging which could be potential therapeutic targets for the prevention of age-related cognitive decline.

## Significance

The fields of immunology and neuroscience have evolved in isolation, partially justified by the view that the brain–blood barrier is impermeable, however, the newly developing science of aging has revealed that chronic, high levels of proinflammatory immune factors accelerate the aging process in the brain. MRI scans identify a decline in cortical volume as a marker for aging. We extracted a cytokine clock (CyClo) that was able to estimate physiological age based on the concentrations of a set of blood proteins that change throughout life. Canonical correlation analysis reveals that the variability in the volume of different functional cortical networks associates differentially with age, sex, and CyClo, suggesting selective vulnerabilities of certain functional networks to circulating levels of immune markers of aging.

Understanding the effects of age is of chief importance as aging is the biggest risk factor of disease (1). This observation has stimulated interest in the identification of biological parameters that change with age. For example, epigenetic changes strongly correlate with the calendar age of an individual, thus providing a readout of a biological clock (2). One of the first biological clocks based on changes in the epigenetic landscape showed that time has a direct effect on the biology of an organism, but on the other hand, its design as a simple readout of time-dependent changes in methylated loci constrains its usefulness in understanding the physiology of aging.

Since then, there have been other clocks developed based on physiological factors that change with age and are themselves directly related to alterations in body homeostasis. An example of such a clock is based on the understanding that systemic chronic inflammation drives the progression of age-related decline and predicts multimorbidity (3, 4). Extracting an age clock based on changes of circulating immune factors contains both a causal and a consequential element of the effects of age. As a result, this clock is less precise in capturing the calendar age but reflects more precisely the age-dependent physiological status of the individual. It provides opportunities to directly interact with the physiological parameters showing deterioration with age and restoring them to youthful status.

Accumulating evidence demonstrates that certain changes in brain structures predict cognitive impairment during aging (5). The areas that seem most affected during aging are those important for learning, memory, and other complex brain functions. In the elderly, communication among neurons becomes less effective, and blood flow to the brain may also decrease. Thus, a rise in inflammatory markers with age (inflammaging) appears to be connected to cognitive decline and mental function, even in aging individuals without clinical pathology (6–9).

Since the blood–brain barrier separates neurons from components of circulating blood, changes in its permeability can affect brain homeostasis (10). For example, brain endothelial cells are sensitive to age-related circulatory cues and could be key to understanding the effects of circulating factors on neuroinflammation, cognitive decline, and neurodegenerative diseases (11). Systemic factors interact with the brain–blood barrier, and age-related changes in brain endothelial cells lead to structural and molecular effects of peripheral signaling molecules (12).

A paradox of neurodegenerative diseases is that they are known to have a localized origin and subsequently spread to other initially unaffected regions, while at the same time, they have been linked to alterations outside the nervous system that should exert their effects broadly over the brain. It is unclear how external systemic changes could lead to selective neuronal vulnerability in the areas of disease onset (13, 14). Yet, reduction in inflammatory pathways and increase in dendritic spines, plasticity genes, neurogenesis, olfactory discrimination, and learning/memory as well as vascular remodeling can be achieved by infusion of blood from young animals into aged ones, thus highlighting the interplay between circulating factors and brain health (15).

Across the span of adult human life, there are observable changes in brain volumes, and these are considered part of the typical nonpathological aging processes in the brain (16). Brain atrophy can be observed longitudinally both in a person and in cross-sectional population measures. In both cases, the rate is just under half a percent of volume per year (17). This change is measurable in both global and regional brain volumes but is not significant at the level of the total intracranial volume (TIV) (17). Interestingly, researchers have noticed that with the progression of age, the variance in the measures of brain volumes increases in healthy individuals and decreases in individuals with Alzheimer’s disease (18). This heterogeneity was also identified at the level of trajectories of brain volume atrophy, with frontal regions being more affected in normal aging and temporal regions leading the atrophy rates in Alzheimer’s disease (19, 20). Moreover, separate patterns of brain atrophy have been observed in different disease etiologies (21). Heterogeneity in gray matter volume (GMV) has been also detected with respect to anatomical brain networks (22). However, there is a distinction between brain atrophy in healthy aging and that involved in neurodegenerative disease. Using precise stereotactic cell counting, Morrison and Hof found that, contrary to Alzheimer’s disease, normal brain aging is accompanied by very little neuronal death (23). This finding suggests that age-specific alterations leading to cortical atrophy are more subtle and likely to occur at the synaptic level and/or extracellular matrix, while neurodegenerative disease could be related to cell death in affected regions. Moreover, diseases that affect the brain have different functional outcomes, e.g., Alzheimer’s disease is predominantly related to memory impairment, while Parkinson’s disease is mainly a motor disorder. Functional regions are also known to express age-related alterations (24, 25).

Here, we explored how age and aging factors relate to the atrophy of subnetworks of cortical gray matter identified by their functional roles. We subdivided the cortical gray matter into 7 regions corresponding to functionally identified networks following the segmentation proposed by Thomas Yeo et al. (26). We thus recognized the following functional networks: visual, somatosensory/somatomotor, dorsal attention, ventral attention, limbic, frontoparietal, and default mode network (DMN). In order to remove the variability related to head size, the volume of each network was normalized against the TIV.

We also derived a cytokine clock (CyClo) based on a LASSO model of the expression levels of 24 circulating blood proteins. Using canonical correlation analysis, we explored the correlations of patient’s age, CyClo, and sex against the volume of the 7 functionally determined cortical networks. We obtained 3 correlation functions that capture correlations between the aging factors and the cortical network volumes.

## Results

### Study Design: Assessing Immune Features and Brain Morphology during Aging.

The study followed 554 (246 men and 308 women) subjects recruited in the Hillblom Aging Network, an observational study of healthy brain aging from the Memory and Aging Center at UCSF. Participant average age was 69 y (range, 47–102 y), and the average visit number was 3 (range, 1–13), with at least a year between visits (cf. *SI Appendix*, Fig. S6 for histogram of intervals between repeat visits). The cohort consisted of healthy individuals and patients with cognitive decline (normal = 476, mild cognitive impairment = 57, and mild dementia = 1). More detailed cohort description is available in the *Materials and Methods* section and other publications (27, 28).

**Fig. 1.**
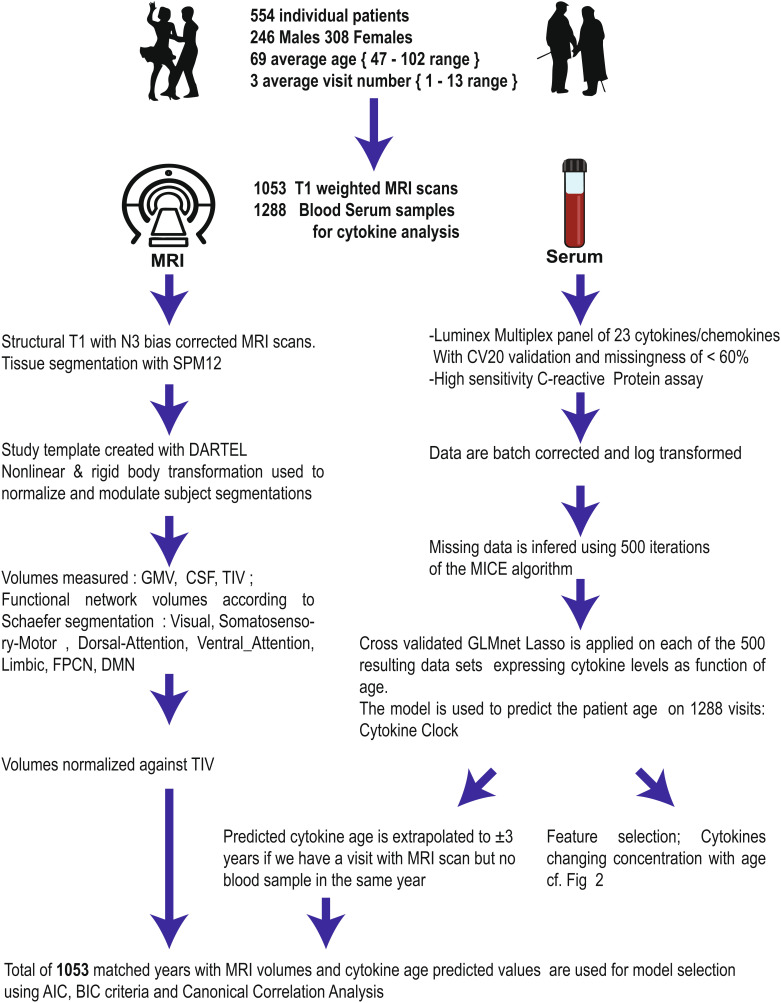
Study pipeline. Blood samples and MRI scans were performed in a population of subjects spanning a 50-y age range. After preprocessing circulating immune proteins, levels were related to age using multivariate linear model with feature selection. This allowed calculation of the physiological age of the patients. The last step analyzes the relationship between calendar age, sex, physiological age, and the morphometric values of functionally determined brain networks.

Fig. 1 summarizes the study design and data treatment pipeline. Subjects underwent structural MRI scans (n = 1,053) and blood serum collection (n = 1,288) performed during yearly study visits. MRI volumes were segmented using the SPM12 unified segmentation procedure, and each patient segmentation was warped using the DARTEL toolbox (29) to create a study-specific template space. TIV and GMV were measured for each patient on the template. Subsequently, the cortical GMV was subdivided into 7 functional networks defined by intrinsic functional connectivity (26). All volumes were measured in milliliters and normalized against the TIV.

Blood serum was collected (n = 1,288), and 24 circulating proteins with potential roles in aging were assayed: TNF_λ_, IFN_γ_, IL-6, IL-10, MCP-1, MCP-4, IP-10, eotaxin, eotaxin-3, VEGF, VEGF-D, VEGF-C, PLGF, bFGF, Vcam-1, Icam-1, Flt-1, TIE2, hs-CRP, SAA, MIP-1a, MIP-1b, TARC, and MDC. Technical constraints to assaying circulating proteins led to some retest samples, with a coefficient of variation (CV) ≥20. The presence of low-reliability replicates means that the values for some proteins in a patient sample must be removed, yielding a dataset with some patient visits having incomplete panel results. We illustrate the overall missingness values for each protein across the 1,288 visits in *SI Appendix*, Fig. S1. A discussion on assaying concentrations of chemokines and cytokines can be found in an earlier publication (28).

To restore a complete dataset, we implemented an imputation protocol that predicted the most likely value for a missing protein in a panel based on the expression levels of all other proteins and the patient’s sex used as experimental design variables. Inherently, making a prediction of a missing value requires choosing from a distribution of plausible values for the variable, thus generating prediction bias. To avoid this data bias inherent to predicting values, we iterated the prediction process 500 times, resulting in 500 data complete sets of circulating protein panels (*Materials and Methods*) and used the data to generate a CyClo. This procedure generated a complete protein panel for each sample. The more general problem of sampling bias is addressed in part by the cv.glmnet algorithm via cross-validation folds between the training data and a test subset of data left out. During the process, individual patients were randomly assigned to and kept in the same data fold to prevent measures of the same patient appearing in the training and testing data.

### Prediction of Aging Using Blood Circulating Immune Proteins.

Expression levels of some blood proteins were previously shown to correlate with patient age and were influenced by a multitude of confounding factors resulting in very noisy individual values that have only limited practicality as predicting factors (30). On the other hand, the power of a panel of circulating proteins could provide strong insight into the overall status of the patient and by their combined effect reveal the biological age of the circulatory system.

**Fig. 2.**
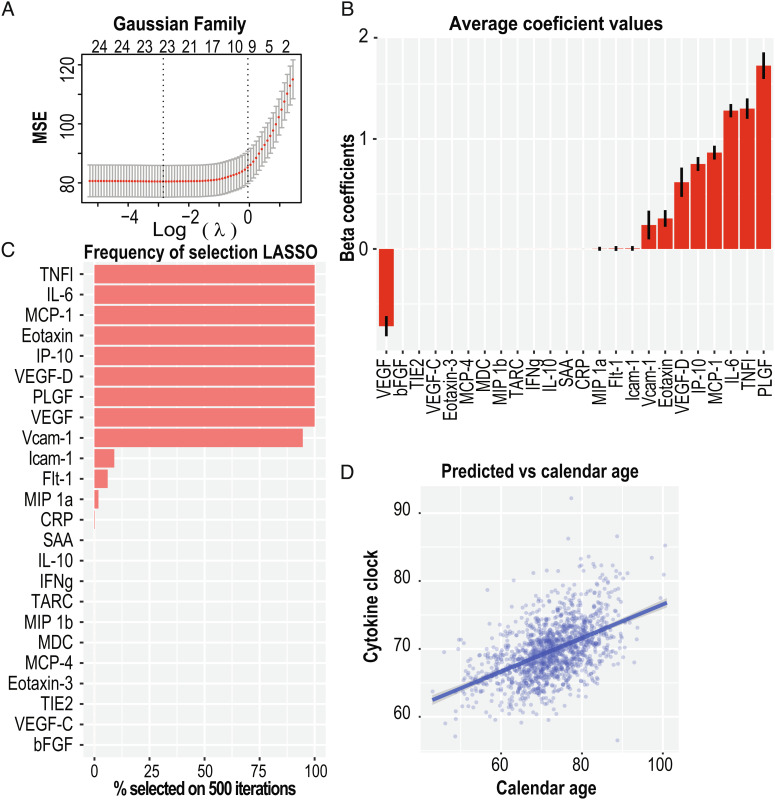
LASSO regression of 24 circulating cytokines against calendar age allows both shrinkage and variable selection. (*A*) The tuning parameter (λ) and variable selection of the LASSO model are chosen via cross-validation. About 14 cytokines are shown to have nonnull coefficients and provide r^2^ within one standard deviation. (*B*) Repeating the procedure for the 500 complete datasets with imputed missing values allows estimation of the average beta coefficients mean and standard error while removing the level of uncertainty introduced by data completion using the mice. (*C*) The frequency at which variables are selected on the 500 runs. Ten parameters have nonnull values for >50% of the iterations. (*D*) Mean values of predicted age for each visit against the actual patient age. Fit is a linear model (adjusted r^2^ = 0.26).

We built a predictive feature selection LASSO model that uses a tradeoff between bias/sparsity against the variance to select a set of proteins whose linear combination optimizes the fit against age. The model uses cross-validation to estimate the optimal value of a penalty factor against feature addition = λ. Fig. 2*A* illustrates the decrease in mean squared error of the model for different values of λ and different numbers of features with nonnull β weights in the linear model. The “unselected” features receive a β value of 0, while the β values of the other features are optimized to maximize the variance. Given that the data are centered and scaled in advance, the β values reflect the information contributed by each feature. Fig. 2*B* shows the average β value for each feature/protein in the 500 datasets. The proteins with the highest contributions are known to play roles in vascular growth (VEGF, VEGF-D, and PLGF), general inflammation (TNF_λ_ and IL-6), chemoattraction for monocytes/macrophages (MCP-1 and IP-10), recruitment of eosinophils into sites of inflammation (eotaxin), and promoting interactions between the vasculature and immune cells (Vcam-1).

We interpreted the set of selected features as a tradeoff between a sparse set of features that provide a less biased model of the variance in the independent variable. In our case, this is the subject’s calendar age. Fig. 2*C* illustrates the frequency at which each feature is selected in the 500 model iterations. There are 9 proteins selected in >90% of the models (TNF l, IL-6, MCP-1, IP-10, Eotaxin, VEGF-D, VEGF, PLGF and Vcam-1). These proteins indicate an optimal set of imputation independent features that minimizes the model error.

Building the LASSO model allows prediction of the dependent variable (subject’s age) based on the levels of the 24 proteins. We used the 500 data complete panels to make an age prediction for each subject. The predicted subject age, which we also call CyClo (cytokine clock), for each subject at a specific visit is the median value of the 500 predictions generated as we trained a LASSO model on each of the 500 data complete cytokine expression panels. Fig. 2*D* displays these predicted ages (CyClo) against the calendar age of each patient at each sample time. The solid “dark-blue” line with a gray-shaded region shows a linear fit of the two variables. The patient’s biological and calendar ages show a moderate correlation of 0.50. We therefore asked whether the CyClo captures additional information beyond the effect of calendar age.

### CyClo Predicts Age-Related Changes in Brain Volumes.

**Fig. 3.**
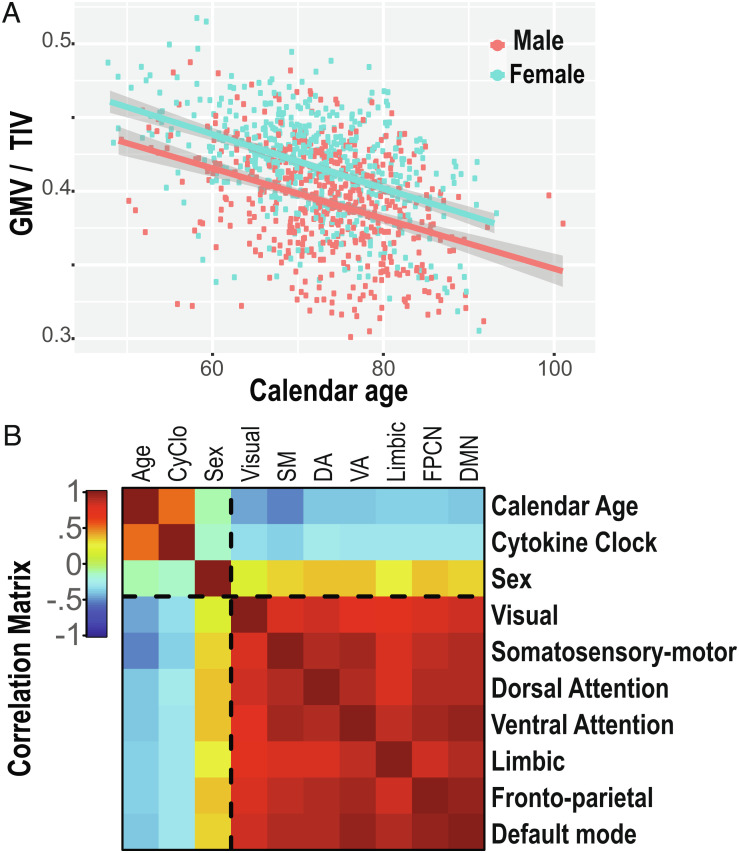
The proportion of gray matter volume to the total intracranial volume is inversely related to calendar and physiological ages (*A*) GMV normalized to TIV against calendar age. Fitted curves are an RLM with sex as a factor. (*B*) Correlation matrix for age, CyClo, sex, and 7 functional brain network volumes.

Changes in brain volumes with age, sex, and relative GMV are well established. Hence, we first investigated GMVs in relation to age and sex in the context of our specific cohort (Fig. 3*A*). Then, we tested whether the linear combination of multiple cytokine levels that we measured contributes significantly to this correlation in addition to the effects of age and sex.

We generated linear mixed-effect models of GMV by incrementally adding explanatory variables and used patients as random effects to account for subject ID. We used the AIC and BIC as penalty criteria for parameter addition. Post hoc χ^2^ ANOVA test between model 3 and model 4 showed significance, with *P* < 0.001***, confirming that the effect of CyClo is highly significant in the presence of the two other variables (age + sex). Table 1 summarizes the results and shows that all 3 parameters (age, sex, and CyClo) contribute significantly to the model robustness. The full model GMV ~ age + sex + CyClo + (1 | subject ID) showed the smallest Akaike information criterion (AIC) of −5201 and Bayesian information criterion (BIC) of −5171. The goodness of fit or r^2^ (R-squared) is poised to increase upon addition of more parameters to the linear mixed-effects model at the risk of overfitting the data and decreasing robustness. The AIC and BIC balance, respectively, against overfitting and underfitting of the model. The full model showed the significant chi-squared ANOVA and lowest AIC and BIC values and is therefore the one model that provides the optimal fit to the data without overfitting. The feature selection criterion showed that all 3 parameters (age, sex, and CyClo) contribute significantly to explaining the variance of GMV.

**Table 1.** ANOVA model evaluation for GMV

GMV ~				
Model	Null	Age	Age + Sex	Age + Sex + CyClo
CyClo2				***
Sex			***	***
Age		***	***	***
Constant	***	***	***	***
AICBIC	−4,875	−5,129	−5,188	−5,201
	−4,860	−5,109	−5,163	−5,171

Parameter selection tests. The linear mixed-effects model with the smallest AIC and BIC score is model 4 that takes into account all 3 parameters (age, sex, and CyClo). ANOVA of models 3 and 4 returns significant *P* < 0.001***.

Random effect = subject ID.

*P < 0.05; **P < 0.01; ***P < 0.001.

To go a step further, we explored the atrophy associated with specific functional networks along human life span. We subdivided the GMV into 7 separate networks of functional connections (visual, somatosensory/somatomotor, dorsal attention, ventral attention, limbic, frontoparietal, and default mode) (26). Fig. 3*B* illustrates pairwise correlations between all resulting variables. All 7 functional networks show overall high correlation among each other. Nevertheless, some structure is obvious, with the visual and limbic network showing less volume correlations with other networks. The somatosensory/somatomotor and dorsal and ventral attention networks seem to form a higher correlated group (average correlation of all volumes 0.86 vs. within-cluster average 0.9), and the DMN together with the frontoparietal network show another pair of highly correlated volumes (correlation of 0.95 vs. the average correlation of all volumes of 0.86). The somatosensory/somatomotor network volume shows the highest correlation with age (R = −0.50 and *P* < 0.01), while overall, the correlation with age and CyClo is between −0.27 and −0.46 with *P* < 0.01. Sex being a binary variable, the correlation level represents the average volume difference between men and women. The visual network volumes seem the most balanced, while the other networks show varied levels of balance between sexes. This finding is consistent with observations from recent publications (31, 32). *SI Appendix*, Fig. S5 illustrates the within-set associations, with the univariate density functions in the diagonal; the lower triangle is a pairwise scatterplot, and the upper triangle shows the corresponding correlation coefficient for the pair and its significance score.

Taken together, these results indicate that the changes in immune proteins with age can be used to accurately predict the age of a person, thus offering insight into the biology of the aging immune system.

### Canonical Correlation Analysis Reveals Age-, Sex-, and CyClo–Specific Changes in Brain Functional Networks.

Our previous analysis illustrated the high within- and between-group correlations among the aging factors and the functionally determined gray matter networks. It also revealed patterns suggestive of more complex interactions. One way to explore the structure between two sets of variables is to perform a canonical correlation analysis. This analysis creates a set of latent (canonical) variates, one for each group of variables, yielding a weighted linear combination of the variables in each set. The weights of the variables generating each variate are optimized so that the correlation between variates is maximized.

**Fig. 4.**
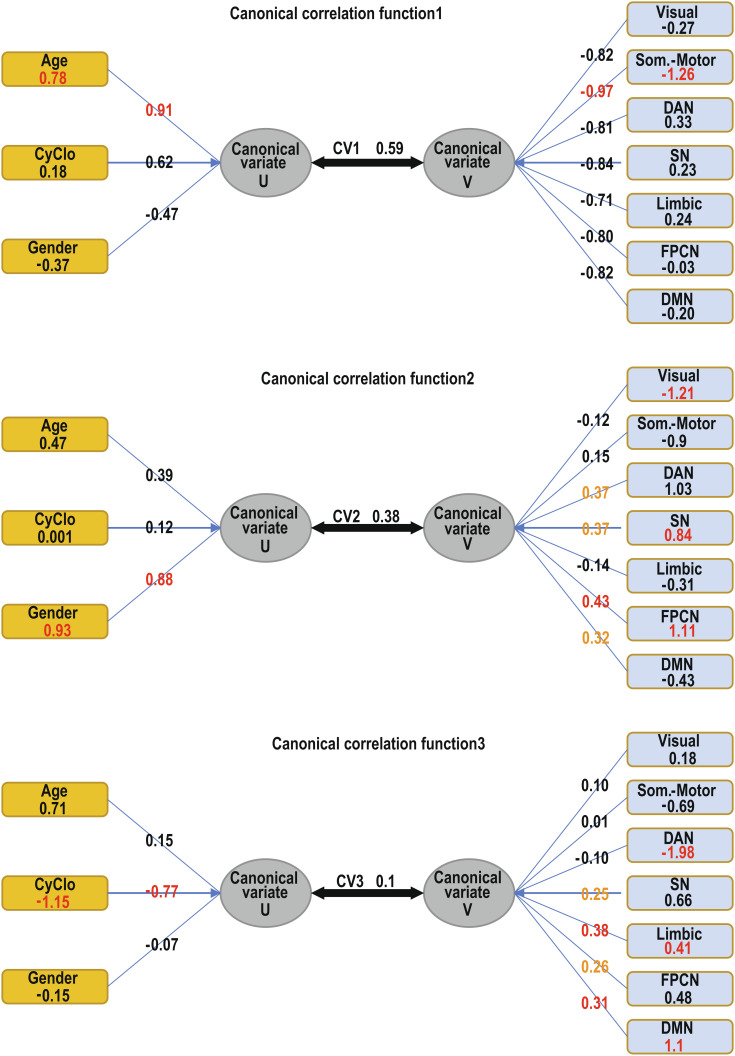
Canonical correlation functions 1–3. The functions illustrate the standardized canonical coefficients (weights) for each variable presented in a rectangular box. The canonical loadings (also called canonical structure correlations) for each variate are listed over the converging arrows, and the canonical correlations on the arrow connect the two ovals representing the canonical variates. In red and yellow are the coefficients of the variables with the highest contribution for each canonical variate. Note that the canonical weights are specific to each canonical function and represent the weighted contribution of each measured variable to the synthetic canonical variate; they can be interpreted within the function. On the other hand, the canonical loadings of each variable can be interpreted as similar to factor loadings and reflect the variance that the observed variable shares with each canonical variate. There is no mathematical reason behind a coincidence of the highest canonical weights and highest loadings in a given function, and it is therefore simply an indication of consistent results.

We conducted a canonical correlation analysis, which generated three canonical functions illustrated in Fig. 4. The figure graphically tabulates the coefficients of the 3 canonical functions. Function 1: The variable with the highest contribution to the variate U_1_ is age (standardized canonical weight 0.78 and canonical loading 0.9) and is reciprocated by the somatomotor system (standardized canonical weight −1.26 and canonical loading −0.97) as the highest contributor to the canonical variate V_1_. This function generates canonical variates with a correlation of 0.59 and covers a shared variance of ρ^2^ = 0.35 (DF = 21 and *P* < 0.001, Bartlett’s χ^2^ test). Function 2: The variable with the highest contribution to the variate U_2_ is sex (standardized canonical weight 0.93 and canonical loading 0.88) and is reciprocated by the frontoparietal (standardized canonical weight 1.11 and canonical loading 0.43), ventral attention (standardized canonical weight 0.84 and canonical loading 0.37), and visual (standardized canonical weight −1.21 and canonical loading −0.12) networks, which present the highest weights to the variate V_2_. This function generates canonical variates with a canonical correlation of 0.38 and covers a shared variance of ρ^2^ = 0.14 (DF = 12 and *P* < 0.001, Bartlett’s χ^2^ test). Function 3: The variable with the highest contribution to the variate U_3_ is the CyClo (standardized canonical weight −1.15 and canonical loading −0.77) and is reciprocated by the default mode (standardized canonical weight 1.1 and canonical loading 0.31), limbic (standardized canonical weight 0.41 and canonical loading 0.38), and dorsal attention (standardized canonical weight −1.98 and canonical loading −0.10) networks as the highest contributors to the canonical variate V_3_. This function generates canonical variates with a canonical correlation of 0.1 and shared variance of ρ^2^ = 0.01 (DF = 5 and *P* ≤ 0.038, Bartlett’s χ^2^ test). All three functions can be considered significant at below 5% threshold and indicate that different combinations of aging factors and functional brain networks help optimize correlations between the two sets.

The canonical correlation coefficients are a symmetrical measure of correlation between the canonical variates U of the set of variables X and the canonical variates V of the set of variables Y. We also calculated the redundancy coefficients that are nonsymmetrical and provide an estimate of the amount of variance in one canonical variate that is explained by the other canonical variate. The redundancy coefficient on X|Y was 0.21 and Y|X was 0.25, which can be interpreted as predictive power, with aging factors having stronger predictive power on the expected functional network volumes than the reciprocal.

Together, these results demonstrate that i) after correcting for age and sex, the CyClo reflects changes in GMV (ANOVA model selection GMV ~ age + sex + CyClo + (1 | subject ID)) and ii) age, sex, and CyClo correlate to different extents with distinct functional networks in the brain (canonical correlation analysis).

## Discussion

Here, we used a systems approach involving different technological platforms to map blood immune biomarkers to age-related morphometric changes in the brain and understand how different factors contribute to volume variation in specific brain areas that comprise functional brain networks. We were able to decouple the effects of age from those of sex and circulating proteins onto these functional networks. We confirmed previous findings associating aging with changes in certain areas of the cortex (somatosensory and somatomotor) and identified specific immune proteins that correlate with changes in the default mode and the limbic and dorsal attention networks, which are areas involved in attention, emotion, memory, and response to social stress, internal evaluation, access to consciousness, and creativity functions. Since brain disorders such as Parkinson’s disease and late-onset Alzheimer’s disease have increased prevalence in older patients and are often considered diseases of “old age” (33–35), our study suggests that modulating the immune system with age can have implications for brain health in a very specific manner, thus dissociating the effect of age from the effects of age-related immune factors.

Together with the skin, the brain might be the one organ where the compound effects of age might be the most obvious at the structural level, thus confounding age as a corollary with other potential factors of atrophy (36, 37). On the other hand, we know that external factors such as diet and behavior, as well as genetic background, play a role in the onset of neurodegenerative disorders (38). Previous studies show that external factors can interact with the aging brain by modulating cytokine levels (39). However, the directionality and specificity of immune proteins affecting different brain regions have not been studied. Both major “age-related” brain pathologies are considered progressive diseases with a localized origin and subsequent propagation to the whole brain (40, 41).

In this study, we distinguished among the effects of age, sex, and circulating blood proteins as reflected in the age-related morphological changes in 7 distinct cortical regions involved in different functional networks. We distinguished the visual, somatomotor, dorsal attention, ventral attention, limbic, frontoparietal, and DMNs and measured the corresponding brain volumes. The choice of working with functionally determined networks that represent functional specializations rather than tracking the anatomical neighborhood allows us to focus on evolutionarily determined units rather than simple topological proximity. This approach is consistent with theories of brain evolution operating by duplication of modules, which could lead to physical proximity without functional similarity (42–45).

The levels of 24 circulating proteins were measured over 1,288 visits in patients with a 50-y age span. Statistical modeling allowed us to identify a combination of 9 proteins that change expression levels with age, and we built a CyClo that predicts age by a weighted combination of the expression levels of these proteins. Previous studies focused on immune proteins to understand how the immune system participates in the development and severity of age-related pathology (46–50). In this study, machine learning approaches showed that this CyClo helps explain the variance in GMV, even if age and sex are accounted for as factors. Thus, we considered changes in all three factors against the brain volumes. Canonical correlation is widely used to identify and measure associations among two sets of variables. In medical problems, canonical correlation has been shown to be applicable to discrimination of multivariate relationships among modalities of the same subject (51–53) and is appropriate in situations where multiple regression would be but where there are multiple intercorrelated outcome variables (54). For example, via canonical correlation, studies have demonstrated that multiple SNPs correlate with multiple disease phenotypes (55) and found genetic variants that correlate with Alzheimer’s disease (56). In brain aging and Alzheimer’s disease, cytokine levels were shown to affect microglia activation in order to reduce amyloid burden as a protective mechanism, but chronic unresolved inflammation leads to pathological outcomes (57).

Our analysis using canonical correlation generated three correlation functions corresponding to the number of parameters in the smaller dataset (age, sex, and CyClo). We chose these functions to optimize the correlation of latent variables generated by weighted linear combinations of the factors of each dataset. On the left-hand side of each function, the latent variable was generated by overweighted contribution from a distinct aging factor (age, sex, or CyClo), while the right-hand side was loaded with a weighted combination of the volumes of the seven functional networks that maximized the correlation. This resulted in a differential participation of different networks in the three functions. The function most heavily loaded with patient’s age information received the highest load from the somatosensory/somatomotor cortex to maximize the correlation, suggesting that age by itself relates mostly to changes in this network. The function that distinguished sex information was reciprocated by strongest participations from the frontoparietal, visual, and ventral attention networks, implying that these networks are more strongly differentiated by sex rather than age or cytokine levels. The function receiving the heaviest weighting from cytokines was reciprocated by heavy participation of the default mode and limbic and dorsal attention networks, indicating that these networks express sensitivity to circulating aging factors. The two main factors we identified to change expression levels with age are VEGF and PLGF. Both play roles in vascular health. A third factor with significant participation in the cytokine age clock is VCAM-1, known to play a role in recruitment and adhesion of immune cells to epithelial cells. Interestingly, infusion with aged blood was found to impair hippocampal neural precursors and activate microglia via VCAM-1. This finding suggests localized effects of circulating molecules (58). Further evidence suggests that the blood–brain barrier is strongly implicated in the pathology of both Alzheimer’s and Parkinson’s diseases (41)(59). Our results are consistent with that view. Furthering the understanding of the interaction between the immune system and the status of the central nervous system (CNS) is an essential step in the quest to understanding neurodegenerative diseases. Accumulating evidence suggests that cell-free proteins in the cerebrospinal fluid correlate with age, and these proteins are associated with inflammation and response to injury in the CNS (60). These observations have been extended to peripheral circulating proteins(61–63), and predictive models of cognitive impairment and dementia have been generated based on plasma proteins (47). Our research focused on a small number of chemokines and cytokines with potential involvement in inflammatory processes and inflammaging, and we found that the atrophy in specific functional networks of the cortical gray matter correlates with these protein concentrations in the peripheral blood. More detailed studies of the proportions of different immune cells in the blood and untargeted proteomics analyses of circulating blood proteins in large cohorts would allow better understanding of how the immune system shapes aging and neurodegenerative diseases (64).

In summary, using an unbiased approach to immunological and brain aging in a large cohort, we have decoupled the effects of age, sex, and inflammaging in brain morphometric features, including functional networks. We find that while age influences somatosensory and somatomotor effector functions, inflammatory mediators correlate with changes in different networks, preferentially affecting brain processes such as focused attention, emotion, memory and response to social stress, internal evaluation, access to consciousness, and creativity. This study suggests that modifying the immune biomarkers found here could have therapeutic implications for preventing brain aging.

## Materials and Methods

### Participants.

The data for this study were derived from the Hillblom Aging Network cohort that was established by the Memory and Aging Center at the University of California San Francisco (UCSF). Participants for this cohort are primarily recruited via community outreach events, flyers, and media advertisements across the Bay Area in California. The research protocols have received IRB approval by the UCSF Committee on Human Research, and the researchers have followed the principles established by the Declaration of Helsinki. Written informed consent was obtained from all included participants. The data collected consisted of comprehensive health examinations, blood draws for cytokine and chemokine assaying, and structural MRI scans. Scripts and data have been deposited at https://www.synapse.org/#!Synapse:syn42844729/files/ (65).

### Data Collection and Statistical Analysis.

All statistical analyses were performed with R (66). Deidentified data, scripts used for analyses, and other relevant documentation will be made available upon reasonable request by qualified researchers interested in replicating our results or performing independent analyses. Such requests should be sent to the corresponding author.

### MRI Data Acquisition and Preprocessing.

All brain MRIs were performed at the UCSF Neuroscience Imaging Center using either a Siemens Trio 3T or Siemens Prisma 3T scanner. Magnetization-prepared rapid gradient-echo (MPRAGE) sequences were used to obtain whole-brain T1-weighted images (TR/TE/TI = 2300/2.98/900 ms, α = 9° and TR/TE/TI = 2300/2.9/900 ms, α = 9°). The field of view was 240 × 256 mm, with 1 × 1 mm in-plane resolution and 1-mm slice thickness and sagittal orientation for both sequences.

Before processing, all T1-weighted images were visually inspected for quality control, and those with excessive motion or image artifacts were excluded. Magnetic field bias was corrected using the N3 algorithm (67). Tissue segmentation was achieved using SPM12’s unified segmentation procedure, and each participant’s gray matter segmentation was warped using the DARTEL (Diffeomorphic Anatomical Registration using Exponentiated Lie algebra) to create a study-specific template (29) (68). Each participant’s native space gray matter segmentation was normalized and modulated via nonlinear and rigid body transformations to study-specific template space. A Gaussian kernel of 4-mm full width half maximum was applied to smooth the images. Transformations (linear and nonlinear) between the DARTEL space and ICBM space were conducted to enable statistical comparisons (69). Each subject’s segmentation was carefully inspected to ensure robustness of the process. Quantification of total gray volume was accomplished by summing all voxels in each subject’s native space segmentation. The TIV was calculated for each subject as the sum of the gray matter, white matter, and cerebrospinal fluid segmentations. For this study, our regions of interests were total gray and white matter volumes. All MRI volumes were expressed in milliliters and then normalized against the TIV.

### Cytokine Assay Collection and Preprocessing.

Under the current state of the art, the quantification of blood-based inflammatory biomarkers, chemokines, and cytokines that have plasma concentrations in the picogram per milliliter range is largely platform dependent. For the Hillblom Aging Network, the concentrations of the biomarkers are assessed by high-performance electrochemiluminescence using the MSD (Rockville, MD) V-PLEX human proinflammatory chemokine and cytokine panels. More detailed explanation of procedures is available in earlier publications from the cohort (70–73). In short, the blood collection is performed in the morning after a 12-h overnight fast. The tubes are centrifuged for 15 min at 4°C at 2,000 × g. The plasma is collected and stored in 500-µL polypropylene cryovials at −80°C until the assay is run. Following the manufacturer’s guidelines, the plasma is gradually raised to room temperature, and aliquots of 25 µL for the proinflammatory panel and 12.5 uL for the chemokine panel are loaded into a 96-well plate and diluted 2× for the proinflammatory cytokine panel and 4× for the chemokine panel. The multiplex arrays are analyzed in the MESO QuickPlex SQ 120 Imager and treated with Discovery Workbench v4.0 software (provided by the MSD). The plasma samples are always measured in duplicate as per the manufacturer’s protocol, and a CV is calculated for each sample's test–retest concentration measurements. The “acceptable” value of CV was set to 20, and all detectable concentrations with a CV≤20 were retained. All procedures were performed by board-certified technicians not familiar with the study purpose.

Several cytokines with missing data in more than 55% of samples were removed from our analysis (IL-1β, IL-4, IL-8, IL-2, IL-12p70, and IL-13). An assay of hs-CRP was added. The high-sensitivity C-reactive protein test (hs-CRP) has the ability to detect general levels of inflammation and has been shown to be indicative of the odds of “healthy aging” (74–76). Circulating blood immunoprotein expression levels were natural log transformed. The blood sample assays were processed in 4 batches. To remove the unwanted batch effects associated with technical variables, we used the “removeBatchEffect” function from the limma (77) package, with participant age and sex accounted for as experimental design parameters. The resulting dataset is visualized in *SI Appendix*, Fig. S1. Of the total 24 markers, 11 had less than 5% missingness and 13 had between 40 and 55%. We imputed the incomplete data by multivariate imputation by chained equations using the R package “mice” (78). "mice" uses a fully conditional specification on a variable-by-variable model and is the preferred multivariate imputation method when it is impractical to specify a joint multivariate distribution of the missing data. It defines a set of conditional densities, one for each incomplete variable, and draws imputations by iterating over these conditional densities. Given that we are focusing on the effects of age on the cytokine levels, we need to work with balanced data with respect to distribution of ages sampled. To avoid overweight contribution of certain age groups, we binned the patient ages and plotted a histogram of the distribution (*SI Appendix*, Fig. S2*A*) and then used the counts of patients in each bin to calculate the regularization weights as the “1/no. of patients per bin” ratio (*SI Appendix*, Fig. S2*B*). The predictor design matrix (*SI Appendix*, Fig. S3) took into account the expression levels of all cytokines and the patient sex. The imputation parameters were set to method = “weighted.pmm” (predictive mean matching), predictor matrix as shown in *SI Appendix*, Fig. S3, and imputation weights determined by the regularization weights as shown in *SI Appendix*, Fig. S2*B*. We ran 500 iterations of the imputation process, thus generating 500 “data complete” cytokine expression sets that we later used to build our CyClo model.

### CyClo.

Each of the 500 datasets were independently modeled, and the outcomes were subsequently pooled to generate results unbiased from the imputation choices. The 500 data complete sets were iteratively fit with a cross-validated multinomial GLM regression using the R package “glmnet” (79) with least absolute shrinkage and selection operator (LASSO) via the cv.glmnet function. The model contains the 24 blood proteins as predictor variables and age as outcome variable. The model family is set to “Gaussian,” and data balancing weights are applied in the same way as described for the imputation process. In short, each sample is weighted by 1/no. of samples in the age bin. The alpha parameter is set to 1 to enforce the LASSO method. In order to obtain comparable beta values, the cytokine data were centered and scaled in advance, so the in-function parameter “standardize” is set to “false.” If this step is omitted, the generated beta values will be dependent on the level of expression of each protein and will not be comparable across proteins. The output of each model fit is generated using the “coef” function that tabulates the beta values of the model and the “predict” function that outputs an estimate age for each subject based on their protein expression levels. We calculated for each patient sample the average value of the 500 predictions and call this value “cytokine age.” We are working with a total of 2,339 visits of which 1,288 have cytokine measures and 1,053 have MRI scans. There are 536 visits where both a blood sample and MRI scan are reported in the same visit. In order to increase the overlap, we propagated the CyClo to neighboring visits with a chosen range of ± 3 y (e.g., a patient only had an MRI scan at her visit in 2019 but she has a visit with cytokine measure in 2020, we will take the 2020 CyClo value of this patient subtract 1 and register it for the visit of 2019). The choice of a 3-y range was made so that it is less than half of the precision of the CyClo itself. With this approach, the outcome is 1053 matched visits with MRI measure and corresponding CyClo estimate.

### Statistical Analysis of GMV, Age, and CyClo.

We analyzed the relative contribution of calendar age, sex, and CyClo in explaining the variance of the GMV measured via MRI (Fig. 3*A* and *SI Appendix*, Fig. S5). We created four linear mixed-effect models incrementally adding independent variables and accounting for subject ID as a random-effect model 1 (null) = GMV ~ (1 | subject ID), model 2 = GMV ~ age + (1 | subject ID), model 3 = GMV ~ age + sex + (1 | subject ID), and model 4 = GMV ~ age + sex + CyClo + (1 | subject ID)). We used the AIC and BIC to evaluate the tradeoff between addition of new parameters and the model goodness of fit expressed by the R-squared. Post hoc chi-squared ANOVA of model 3 and model 4 returned a *P* < 0.001***, suggesting that the CyClo parameter is significant in the presence of the two other variables in a random intercept model. The results are summarized in Table 1. Once we established the relation between the overall GMV and the patterns of cytokine aging, we split the GMV into 7 functionally defined subnetworks and then computed the correlation between all variables. This is illustrated by the correlation matrix in Fig. 3*B*.

### Canonical Correlation between Brain Volumes and Age Parameters.

In order to identify the multiparameter correlations between CyClo, age, sex, and cortical gray matter subdivided into a set of 7 functional networks, we performed a canonical correlation analysis as implemented by the CCA package (80) and the yacca package (81). We created two, line-matched matrices, matrix X (calendar_age, cytokine_age, and sex) and matrix Y (visual, somatosensory/somatomotor, dorsal_attention, ventral_attention, limbic, frontoparietal, and default_mode). Canonical correlation analysis is a general case of multivariate regression analysis; it generates functions of latent (canonical) variables U and V that maximize the correlation between the two sets of multiple variables X and Y (82). The new synthetic variables are called canonical variates ${\mathrm{CV}}_{U1}=a_{1}x_{1}+a_{2}x_{2}...a_{p}x_{p}$ and ${\mathrm{CV}}_{V1}=b_{1}y_{1}+b_{2}y_{2}...b_{q}y_{q}$. Together, a pair of canonical variates forms a canonical function. The first canonical function is chosen in such a way that the linear combination of the weights ($a_{1},a_{2}...a_{p};\;b_{1},b_{2}...b_{q}$) of all variables maximizes the amount of correlation explained. The subsequent functions are calculated on the residuals of the previous function and thus finding linear combinations that are uncorrelated with the first canonical correlation. The maximum of canonical functions is equal to the number of different variables in the smaller set, but the number of significant canonical functions can be less than that. In our case, the smallest set of variables contains three variables, and therefore, we can have at most 3 canonical functions. The canonical correlation coefficient is the amount of correlation between the canonical variates in a function. We calculated the 3 canonical functions with the “cc” function and extracted the loadings with the “comput” function. Fig. 4 illustrates the 3 canonical functions, the respective variates, coefficients of variation, the regularized canonical weights or coefficients of each variable, and the loadings of the variable in the function. We tested the significance of each dimension of the canonical variates using Rao’s F approximation and Bartlett’s chi-squared test. It performs Rao’s test and provides statistics on 3 tests. Test 1 informs whether all 3 canonical functions together are significant. Test 2 checks the significance of dimensions 2 and 3 together. Test 3 checks if the third dimension is significant by itself. It calculates *P* values using F approximations of the test statistics of number of observations, size X, and size Y, and the input canonical correlation coefficients are squared to generate the canonical roots (ρ^2^) that capture an estimate of the shared variance.

## Supplementary Material

Appendix 01 (PDF)

## Data Availability

The data collected consisted of blood draws for cytokine and chemokine assaying, and structural MRI scans. Scripts and data have been deposited at Synapse (https://www.synapse.org/#!Synapse:syn42844729/files/) (65). De-identified data and the scripts used for analyses, and other relevant documentation will be made available upon reasonable request by qualified researchers interested in replicating our results or performing independent analyses. Such requests should be sent to the corresponding author.
