# Supplementary material for: Age-related brain atrophy is not a homogenous process: Different functional brain networks associate differentially with aging and blood factors
Source: Proc Natl Acad Sci U S A. 2022 Dec 2;119(49):e2207181119. doi: 10.1073/pnas.2207181119 (PMC9894212; doi:10.1073/pnas.2207181119)
Supplement: Supplementary file 1 — Appendix 01 (PDF) [file pnas.2207181119.sapp.pdf]

PNAS
www.pnas.org

Supplementary Information for

Age related brain atrophy is not a homogenous process: different functional brain networks associate differentially with aging and blood factors

Nikola T. Markov[1], Cutter A. Lindbergh[2,7], Adam M. Staffaroni[2], Kevin Perez[1,3], Michael Stevens[1], Khiem Nguyen[1,6], Natalia F. Murad[1], Corrina Fonseca[2], Judith Campisi[1], Joel Kramer[2] and David Furman[1,4,5*]

[1]Buck AI Platform, Buck Institute for Research on Aging, Novato, CA, USA
[2]University of California, San Francisco, Weill Institute for Neurosciences, Memory and Aging Center, Department of Neurology, San Francisco, CA, USA
[3]University of Lausanne, Lausanne, Switzerland
[4]Instituto de Investigaciones en Medicina Traslacional (IIMT), Universidad Austral, CONICET, Pilar, Argentina
[5]Stanford 1000 Immunomes Project, Stanford University School of Medicine, Stanford, CA, USA
[6] NTT Hi-Tech Institute, Nguyen Tat Thanh University, Ho Chi Minh City, Vietnam
[7] Department of Psychiatry, University of Connecticut School of Medicine, Farmington, CT, USA
***Corresponding author**: David Furman
Email: DFurman@buckinstitute.org

This PDF file includes:

Figures S1 to S6
Table ST1

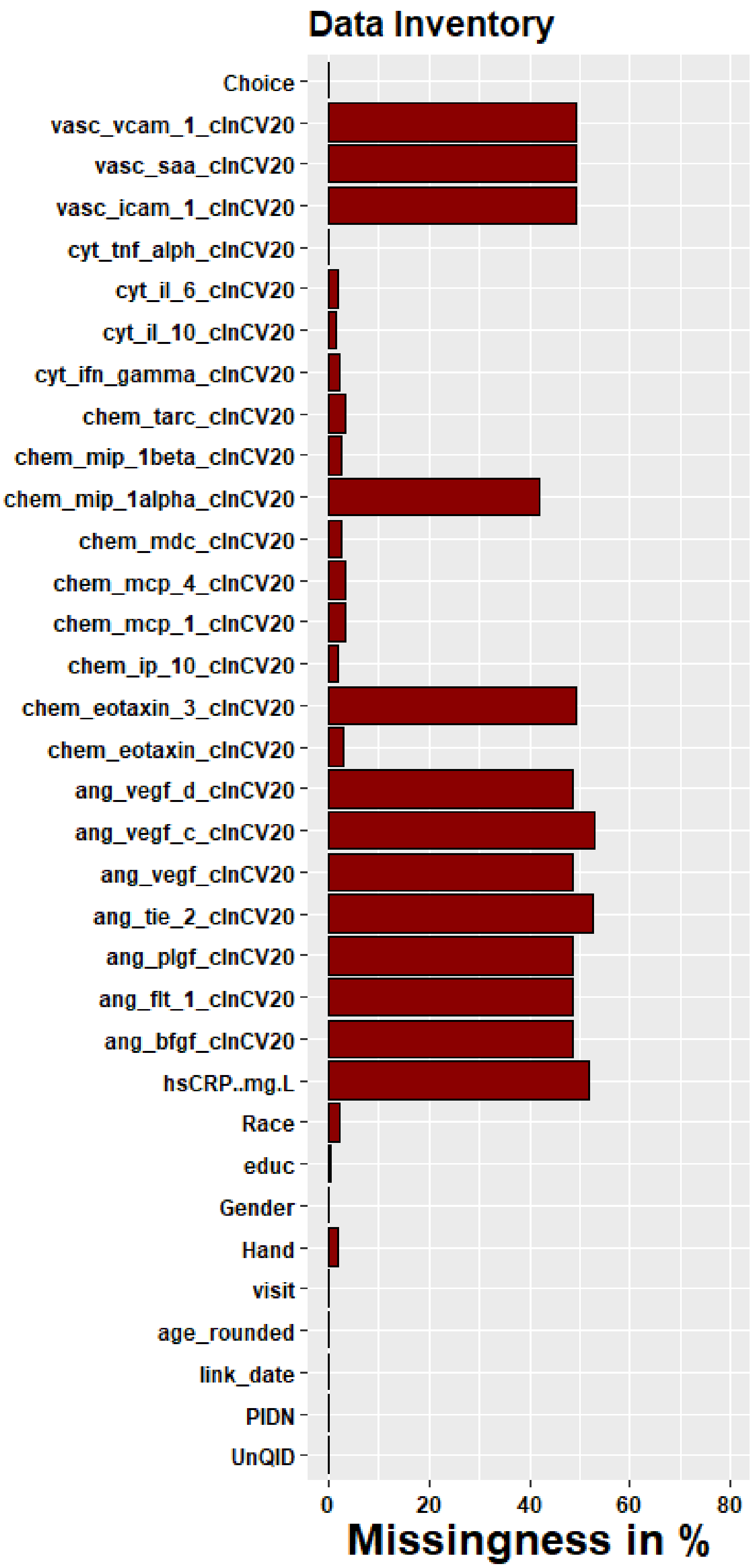

Fig. S1. Data inventory of 1288 visits with cytokine data collection.

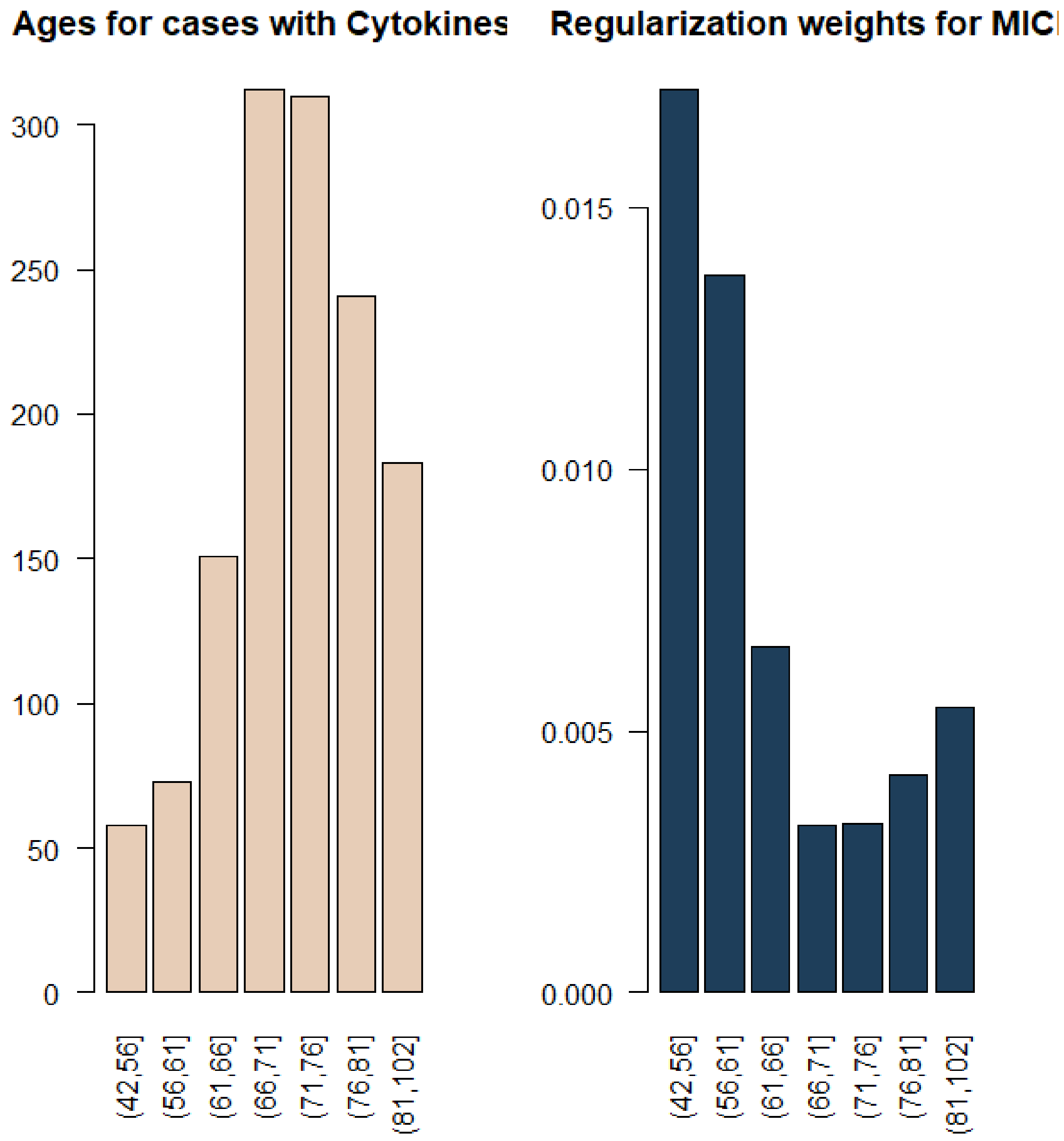

Fig. S2. Distribution of visitation ages and regularization weights based on this distribution.

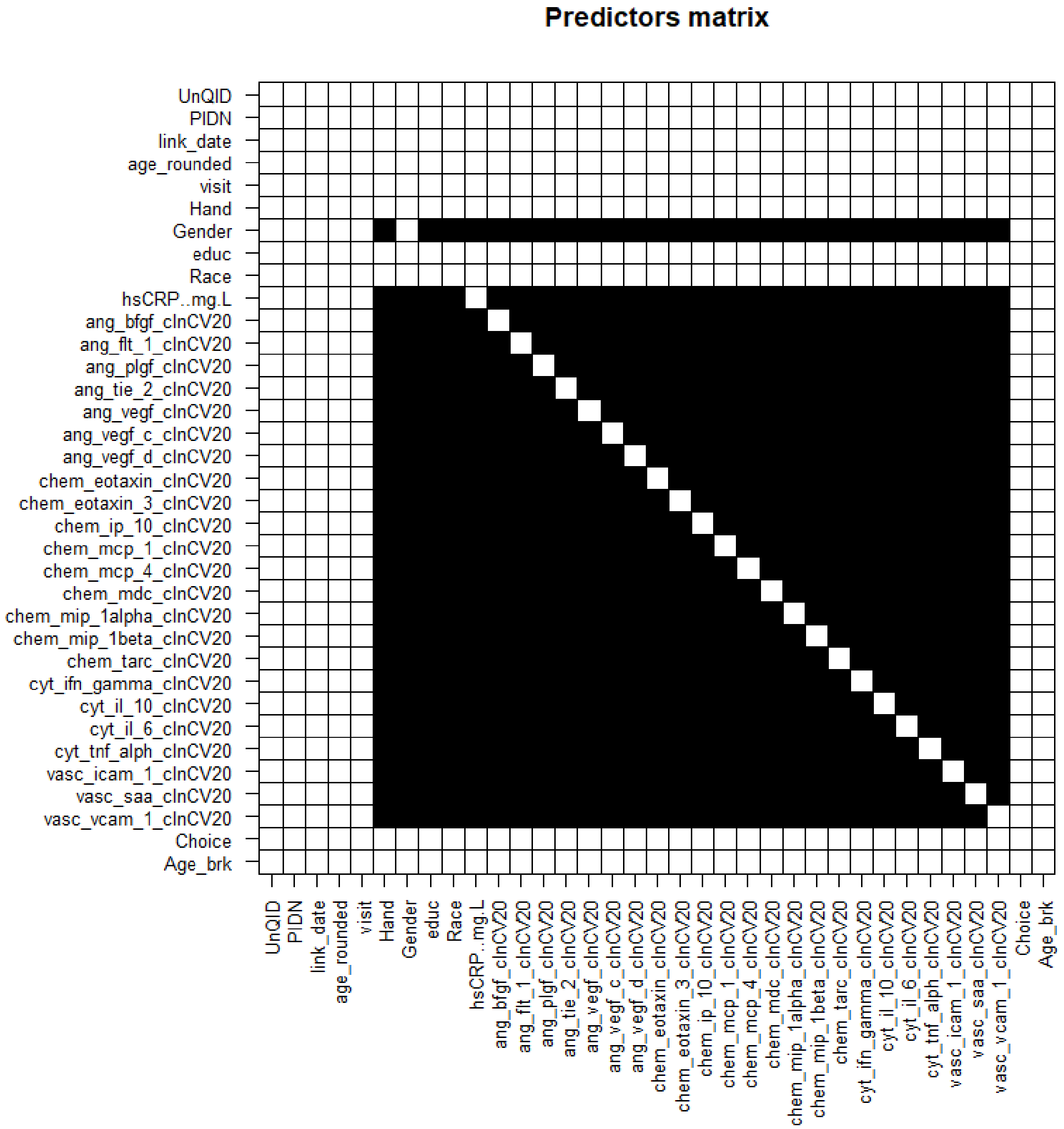

Fig. S3. Predictor matrix used in the generation of the 500 “data complete” circulating protein panels.

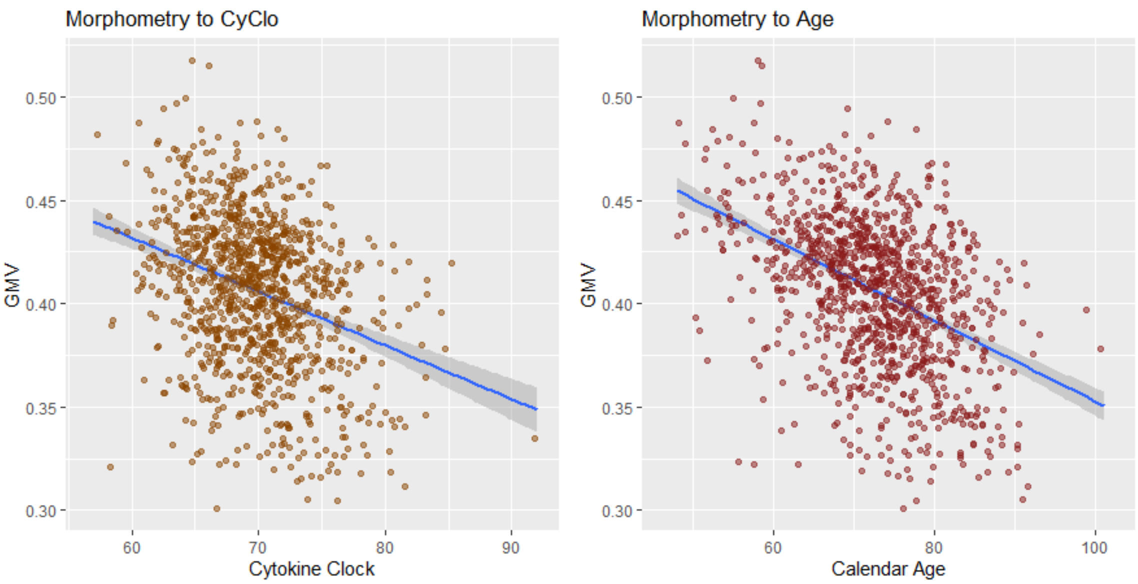

Fig. S4. Linear Regresssion of Gray Matter Volume (GMV) against the Cytokine Clock and Calendar Age.

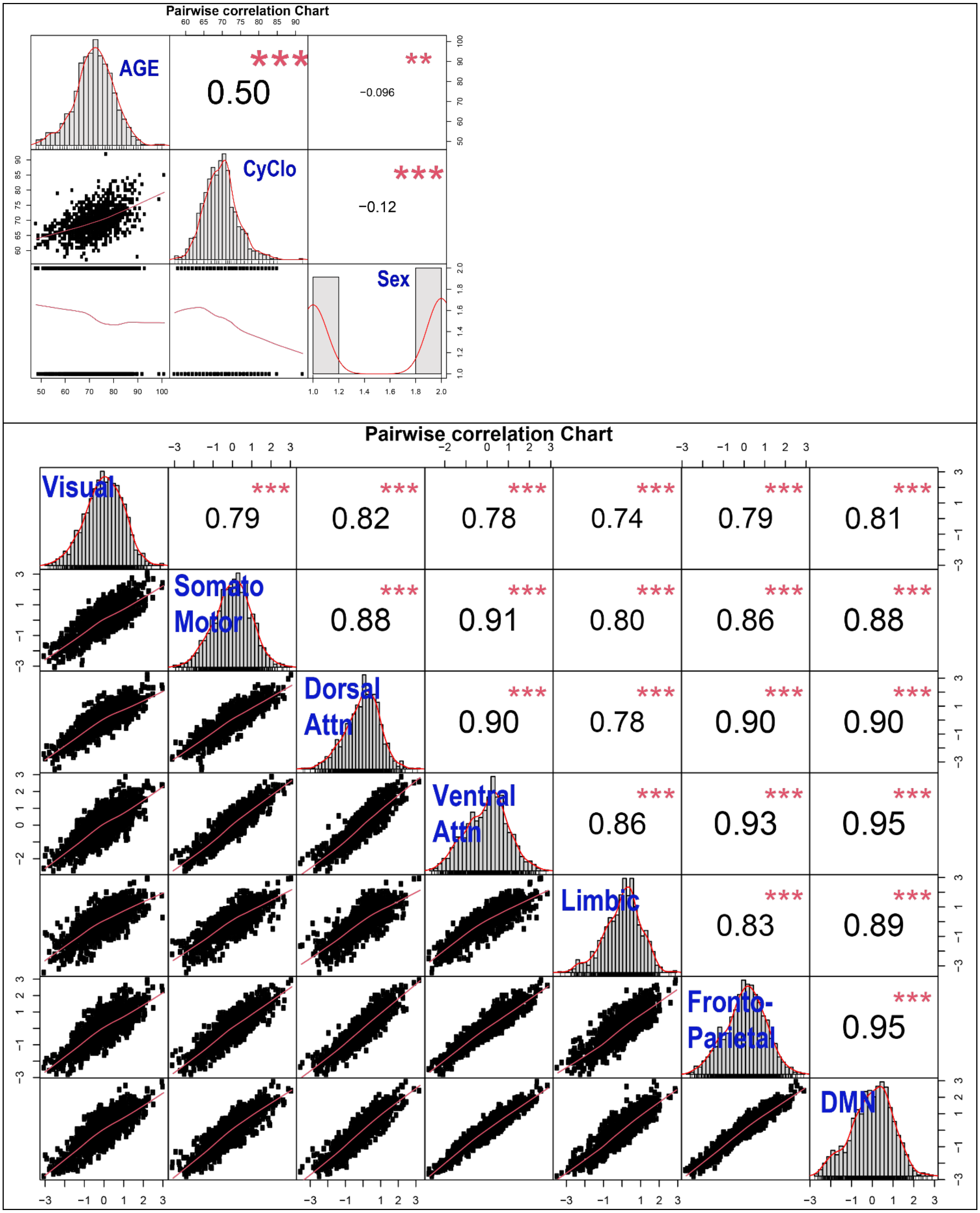

Fig. S5. Pair plots (lower triangle) and Paired correlation test (upper triangle), numbers give R and stars indicate significance of p-val.

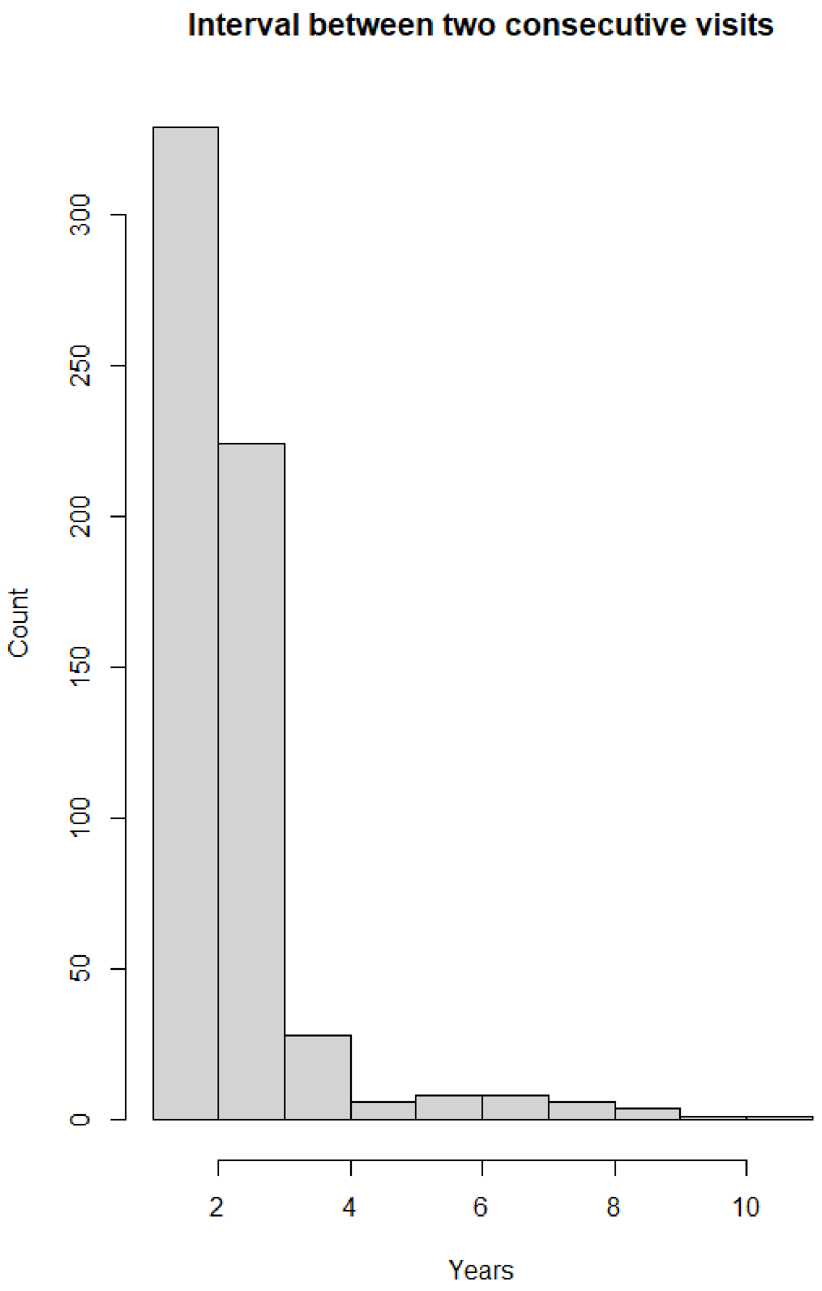

Fig. S6. Histogram plot of the interval between repeated visits for all patients.

Canonical correlation Analysis summary

Canonical Correlations:

CV1	CV2	CV3
0.59	0.38	0.11

Shared variance on each Canonical Variate:

CV1	CV2	CV3
0.35	0.14	0.01

Bartlett's Chi-squared Test:

	rho^2	Chisq	df	Pr(>X)	
CV1	0.35	617	21	<0.001	***
CV2	0.14	170	12	<0.001	***
CV3	0.01	11	5	0.038	*

Canonical Variate Coefficients:

Y vars:	CV1	CV2	CV3
Age	0.7824	0.4742	0.7092
CyClo	0.1848	0.0014	-1.1465
Sex	-0.3688	0.9268	-0.1494

X vars:	CV1	CV2	CV3
Visual	-0.2685	-1.2097	0.1765
Somatosensory/Motor	-1.2561	-0.8857	-0.6888
Dorsal Atthention	0.3336	1.0398	-1.9818
Ventral Attention	0.2267	0.8406	0.6670
Limbic	0.2448	-0.3163	0.4093
Fronto-Parietal	-0.0285	1.1108	0.4846
DMN	-0.2035	-0.4356	1.0966

Structural Correlations (Loadings):

Y vars:	CV1	CV2	CV3
Age	0.9106	0.3861	0.1472
CyClo	0.6241	0.1239	-0.7715
Sex	-0.4668	0.8812	-0.0742

X vars:	CV1	CV2	CV3
Visual	-0.8188	-0.1174	0.1013
Somatosensory/Motor	-0.9749	0.1583	0.0145
Dorsal Atthention	-0.8100	0.3744	-0.1024
Ventral Attention	-0.8350	0.3744	0.2502
Limbic	-0.7106	-0.1449	0.3815
Fronto-Parietal	-0.8016	0.4324	0.2588
DMN	-0.8170	0.3221	0.3106

F test for Canonical Correlations (Rao's F Approximation)

	Corr	*F*	*Num df*	*Den df*	*Pr (>F)*	
CV1	0.59	32.5	21	2995	<0.001	***
CV2	0.38	14.8	12	2088	<0.001	***
CV3	0.11	2.37	5	1045	0.038	*

Table ST1. Summary results of the canonical correlation analysis
